# Nudging healthcare professionals in clinical settings: a scoping review of the literature

**DOI:** 10.1186/s12913-021-06496-z

**Published:** 2021-06-02

**Authors:** Anita Sant’Anna, Andreas Vilhelmsson, Axel Wolf

**Affiliations:** 1 Viniam Consulting, Halmstad, Sweden; 2 Centre for Person-Centred Care (GPCC), University of Gothenburg, Box 100, 40530 Gothenburg, SE Sweden; 3 Institute of Health and Care Sciences, Sahlgrenska Academy, University of Gothenburg, Gothenburg, Sweden

**Keywords:** Nudging, Behaviour change, Decision architecture, Healthcare professionals, Intervention

## Abstract

**Background:**

Healthcare organisations are in constant need of improvement and change. Nudging has been proposed as a strategy to affect people’s choices and has been used to affect patients’ behaviour in healthcare settings. However, little is known about how nudging is being interpreted and applied to change the behaviour of healthcare professionals (HCPs). The objective of this review is to identify interventions using nudge theory to affect the behaviour of HCPs in clinical settings.

**Methods:**

A scoping review. We searched PubMed and PsycINFO for articles published from 2010 to September 2019, including terms related to “nudging” in the title or abstract. Two reviewers screened articles for inclusion based on whether the articles described an intervention to change the behaviour of HCPs. Two reviewers extracted key information and categorized included articles. Descriptive analyses were performed on the data.

**Results:**

Search results yielded 997 unique articles, of which 25 articles satisfied the inclusion criteria. Five additional articles were selected from the reference lists of the included articles. We identified 11 nudging strategies: accountable justification, goal setting, suggested alternatives, feedback, information transparency, peer comparison, active choice, alerts and reminders, environmental cueing/priming, defaults/pre-orders, and education. These strategies were employed to affect the following 4 target behaviours: vaccination of staff, hand hygiene, clinical procedures, prescriptions and orders. To compare approaches across so many areas, we introduced two independent dimensions to describe nudging strategies: synchronous/asynchronous, and active/passive.

**Conclusion:**

There are relatively few studies published referring to nudge theory aimed at changing HCP behaviour in clinical settings. These studies reflect a diverse set of objectives and implement nudging strategies in a variety of ways. We suggest distinguishing active from passive nudging strategies. Passive nudging strategies may achieve the desired outcome but go unnoticed by the clinician thereby not really changing a behaviour and raising ethical concerns. Our review indicates that there are successful active strategies that engage with clinicians in a more deliberate way. However, more research is needed on how different nudging strategies impact HCP behaviour in the short and long term to improve clinical decision making.

**Supplementary Information:**

The online version contains supplementary material available at 10.1186/s12913-021-06496-z.

## Background

Over the last two decades, behavioural economics has gained momentum among scholars because of its innovative but also controversial ways of explaining processes and mechanisms underpinning individuals’ judgements and decision making. It was with their seminal book entitled *Nudge: Improving decisions about health, wealth, and happiness* in 2008 that the behavioural economists Richard Thaler and Cass Sunstein [1] coined the concept of nudging, which they define as: “any aspect of the choice architecture that alters people’s behaviour in a predictable way without forbidding any options or significantly changing their economic incentives.” This means that any limitation of choices, such as banning or withholding information or change in incentive structures like financial rewards or taxation are not considered nudging.

Nudging quickly gained ground in several countries as a new and better method to change people’s behaviour in order to improve their health and well-being [2]. Both private and public institutions showed interest in the use of nudges because they generally cost little and have the potential to promote economic and societal goals, such as public health [3]. Since the origin of the concept in 2008, governments in the US, UK, France and many more have implemented departments of behavioural economics [4–7], sometimes called “nudge units”. Today, there are more than 200 different nudge units globally [8]. The promise of these nudge units is to use the findings of behavioural and social sciences to improve the effectiveness of government policies for modest costs and with little effort [9].

### Nudging theory

In their work, Thaler and Sunstein [1] reference two modes of thinking: the automatic system and the reflective system. Cognitive scientist Daniel Kahneman refers to these as System 1 and System 2 respectively [10]. In System 1, thinking, impressions, associations, feelings, intentions, and actions flow effortlessly and quickly. We are usually in this mode when we go about our daily tasks like brushing our teeth or getting to work. In contrast, System 2 thinking is slow, effortful, and deliberate. This mode is at work when we complete a tax form or learn something new. In psychology, this is referred to as Dual Process Theory (DPT) [11].

DPT is the foundation of nudging because it explains what happens when we act unaware. At the same time, it provides us with the possibility to either make that action salient to ourselves, engaging System 2, or change the context so we choose something better without thinking about it, thus System 1 [12].

### Nudging and public services

While the initial wave of nudging studies targeted mainly the adoption of healthier behaviours during exercise [13, 14], eating [15–17] and quitting smoking [18], nudging is now increasingly being perceived as a policy strategy to improve public services [5, 19–21] like the healthcare system [22].

Healthcare systems are however notorious for their complexity and conservative culture making change management rather challenging [23]. Because of its complexity, change management in healthcare requires more nuanced and well-thought-out interventions instead of top-down strategies, such as issuing more policy, prescribing more regulation, and introducing more stringent performance indicators [24]. While nudging has been increasingly discussed and used for patients and citizens, there has been less focus on the use of nudging strategies targeting healthcare professionals (HCPs).

### Objectives

Our objective was to identify interventions using Thaler and Sunstein’s nudge theory to affect the behaviour of HCPs in clinical settings, focusing on target groups, nudging techniques, delivery systems and empirical evidence. In particular, we were interested in mapping which target groups have been considered, which specific nudging techniques have been used, how nudging strategies have been delivered, whether these strategies present enough empirical evidence of success, as well as the ethical implications of these strategies.

## Methods

A scoping review was conducted from September 2019 to February 2020 according to the Joanna Briggs Institute Reviewers’ Manual 2017 - Guidance for conducting systematic scoping reviews [25].

Scoping reviews are useful for identifying knowledge gaps and synthesise available evidence and can be used to map the key concepts underpinning a research area as well as to clarify working definitions, and/or the conceptual boundaries of a topic [26]. In contrast to systematic reviews, scoping reviews provide an overview of the existing evidence, regardless of quality [25]. We therefore employed a scoping review to map out the ways in which nudging interventions have been used to affect the behaviour of healthcare professionals in clinical settings. The protocol used is explained in the following sections.

### Inclusion criteria

Studies eligible for inclusion were: (i) interventions conducted in a clinical setting targeting healthcare personnel, (ii) behavioural interventions using the term nudging, (iii) randomized controlled trials, quasi-experimental or longitudinal (before-after) studies, (iii) original research articles published in English and in peer reviewed journals between 2010 and 2019.

### Information sources and search strategy

A search for articles was conducted by an information specialist at the university library on two electronic databases: PubMed and PsycINFO. We searched for the term “nudging”, “nudges” or “nudge” in the title or abstract of articles published from 2010. The complete search strategy is provided as supplementary material.

### Article selection

Title and abstract screenings were carried out by two reviewers with the web-based application Rayyan [27]. This screening tool allowed the authors to do individual blinded screenings of titles and abstracts. The selection of studies was undertaken in three phases of screening: (a) removing duplicates, (b) screening titles and abstract, and (c) screening full text articles. At each phase, the articles were compared against the inclusion and exclusion criteria mentioned above. In the case of the conflicting eligibility decisions a third reviewer was asked to give an additional opinion. In all these cases, consensus was finally reached. The reference list of included articles was searched for additional articles that fulfilled the inclusion criteria but that had not been found in the initial search because they did not mention “nudging” in the title nor abstract.

### Data items and charting

A data-charting form was jointly developed by two reviewers to determine which variables to extract. The two reviewers independently charted the data, discussed the results, and continuously updated the data-charting form in an iterative process [28]. The form had the following sections: (a) author(s), (b) publication year, (c) study purpose/objectives, (d) study population and sample size, (e) study design, (f) effect logic, (g) type of nudge used, (h) implementation medium (e.g. physical, digital), (i) implementation details (e.g. clinic, hospital, emergency, etc), (j) geographical location, (k) study duration, (l) main outcomes and/or findings.

## Results

### Article selection

**Fig. 1:**
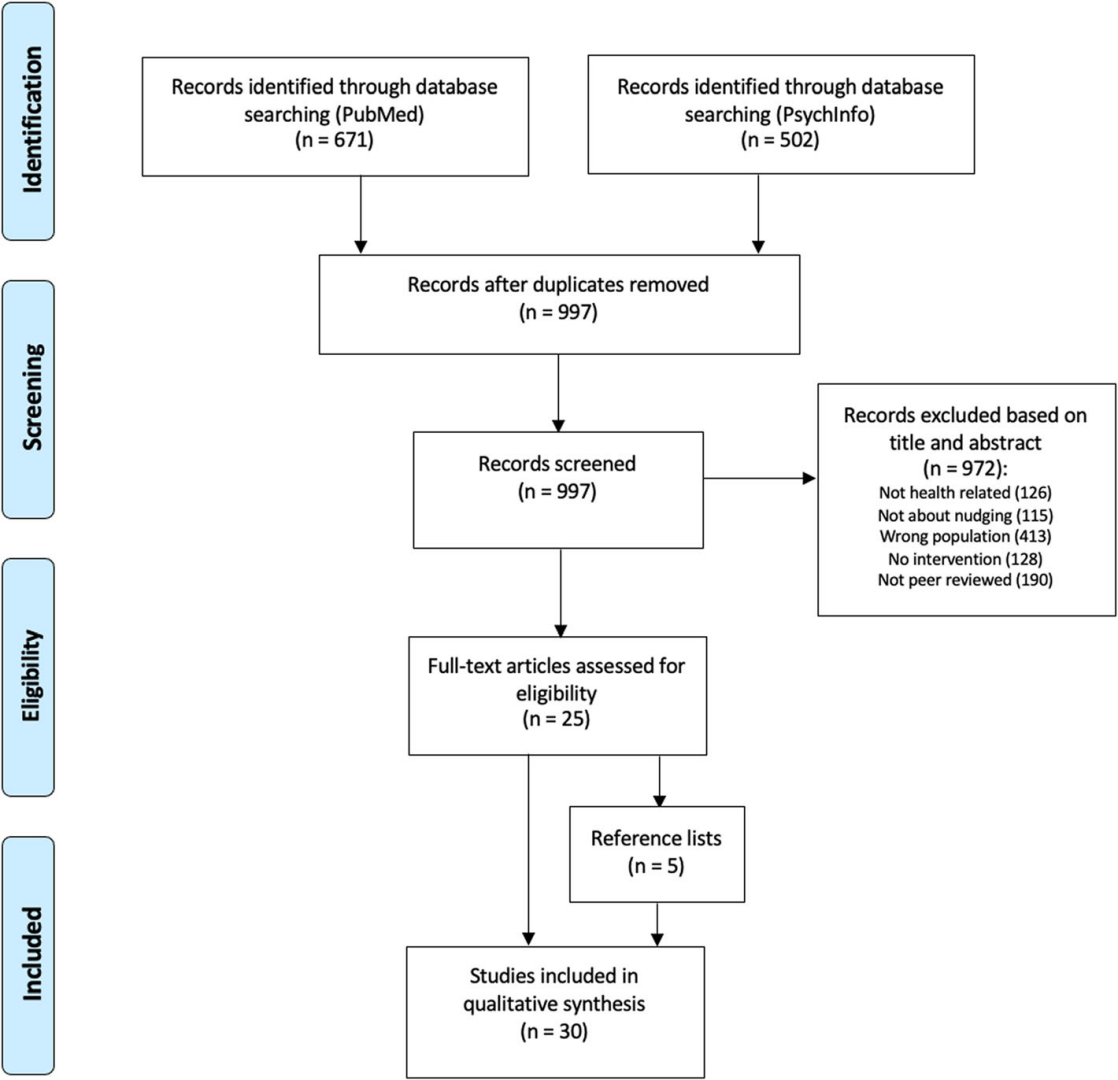
Flowchart of the literature search

**Fig. 2:**
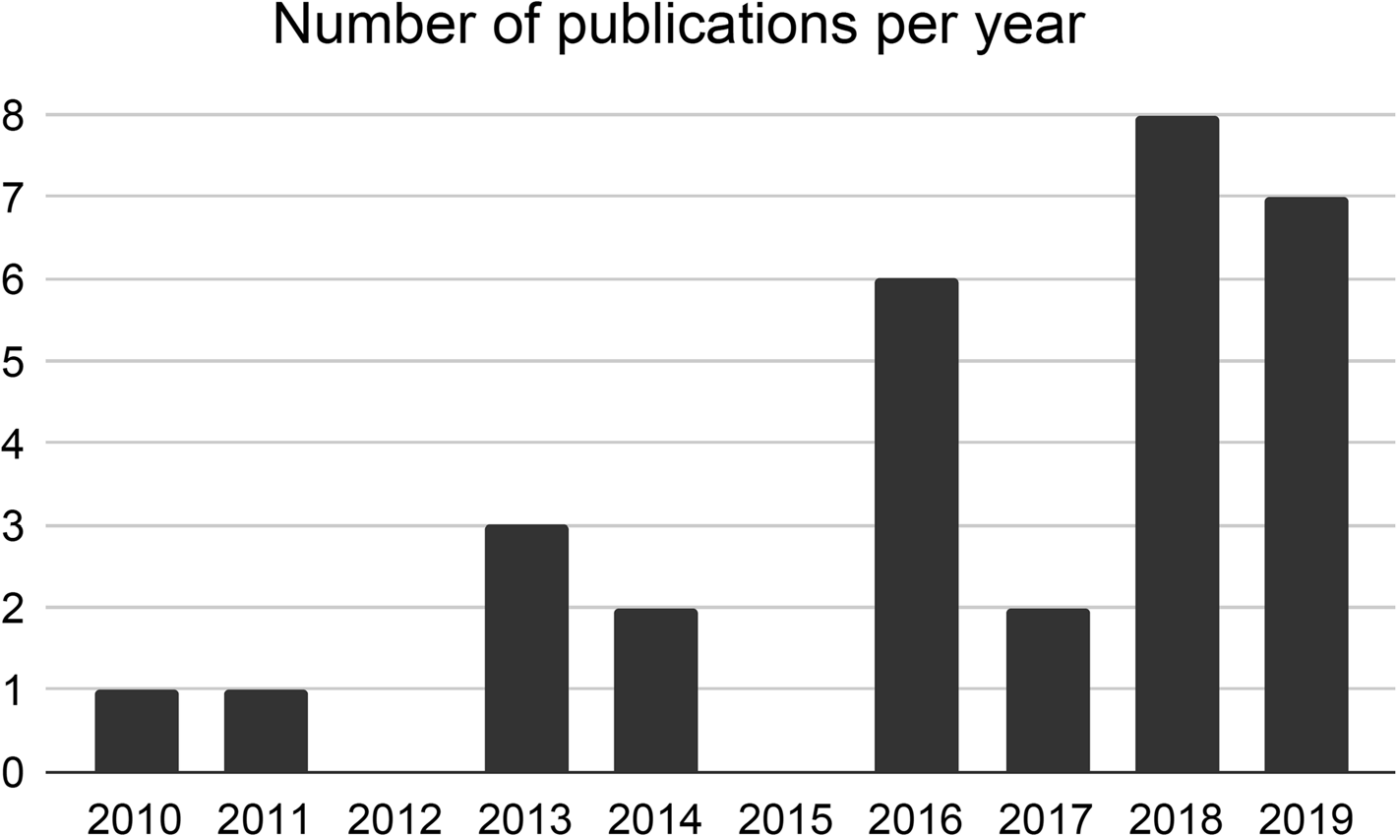
Number of publications per year, 2010–2019

The database searches yielded 1024 articles as shown in Fig. 1. After removal of 27 duplicates, 972 articles were excluded on the basis of not fulfilling inclusion criteria, resulting in 25 full-text articles for inclusion. From the reference lists of these articles, 5 additional articles were identified for inclusion, resulting in a total of 30 articles for analysis. As shown in Fig. 2, the majority of included records (*N* = 23) were published in the last 4 y (2016–2019).

### Study designs

Of the 30 included publications, the majority were prospective studies (*n* = 24) while the remaining six studies were retrospective. Approximately half employed a randomized control group, of which 13 were randomized controlled clinical trials and three randomized vignette-based studies, where clinicians were presented with randomly assigned hypothetical scenarios. One of the randomized controlled trials was undertaken in a simulated environment [29].

Five studies employed a control group without randomization, of which four were pre-post studies that observed a control cohort during the same period as the intervention cohort. The remaining nine articles were pre-post studies without a control cohort (see Table 1). Three of the pre-post studies employed a time-series analysis, comparing temporal trends as well as outcome differences before and after the intervention [30–32].

**Table 1:** Summary of study designs

Study type	Randomised controlled	Controlled	Not controlled	Total
Parallel cohorts	16	1	0	17
Pre-post	0	4	9	13
**Total**	**16**	**5**	**9**	**30**

### Summary of included studies

Table 2 summarises the characteristics of the included studies. The complete data set is available in supplementary materials. Half of the studies were set across hospitals and tertiary care centres and targeted a diverse population of HCPs (physicians, nurses, midwives, medical assistants, novice health care providers, entire clinical team). Eleven studies were set in the context of ambulatory care. One study covered both primary care and specialty clinics, and one study covered an entire ambulatory healthcare system. The remaining four studies were conducted outside clinical environments: one study recruited novice healthcare providers affiliated with a medical school to conduct an RCT in a simulated setting [29], one study recruited practitioners from a collaborating hospital to undertake a vignette-based study [33], and two studies recruited practitioners across geographical regions, namely the greater Chicago area [34] and across Australia [35] to undertake vignette-based studies.

**Table 2:** Characteristics of the articles included in the review

Author/year/ reference	Country	Design	Objective	Setting	Nudging strategies	Nudging medium	Statistically significant positive results?
Birnbach et al. 2013 [29]	USA	RCT (simulated environment)	Improve hand hygiene compliance	Novice health care providers from collaborating medical school	Environmental cueing/priming	Physical environment	Yes
Bourdeaux et al. 2016 [30]	England	Prospective controlled pre-post study (time series analysis)	Increase use of low TVe ventilation in ICU	Tertiary care ICU unit	Defaults/pre-orders; Alerts/reminders	Medical device/machine; Electronic dashboard	Yes
Lewis et al. 2019 [31]	England	Prospective controlled pre-post study (time series analysis)	Reduce unnecessary CT usage	2 hospitals	Environmental cueing/priming	Test results	Yes
Langley et al. 2018 [32]	England	Retrospective pre-post study (time series analysis)	Evaluate whether providing information on the cost of drugs to clinicians would modify total expenditure	Acute medical hospital	Information transparency	EHR	No
Probst et al. 2013 [33]	USA	Randomized vignette-based study	Study the effect of opt-in, opt-out, and recommended order sets on laboratory orders	Physicians at a collaborating hospital	Defaults/pre-orders	EHR	Yes
Tannenbaum et al. 2014 [34]	USA	Randomized vignette-based study	Improve guideline concordance for acute respiratory infection treatment	GPs in the greater Chicago area	Defaults/pre-orders	EHR	Yes
Soon et al. 2018 [35]	Australia	Randomized vignette-based study	Improve guideline concordance for lower back pain treatment	GPs across Australia	Defaults/pre-orders	EHR	Yes
Meeker et al. 2014 [36]	USA	RCT	Increase compliance with antibiotic prescription guidelines	5 primary practice clinics	Environmental cueing/priming	Physical environment	Yes
Yadav et al. 2019 [37]	USA	RCT	Improve guideline concordance for acute respiratory infection treatment	5 Emergency Departments and 4 Urgent Care Centers	Peer comparison; Environmental cueing/priming,	Physical environment, Email	Yes
Caris et al. 2018 [38]	The Netherlands	Prospective pre-post study	Improve hand hygiene compliance	Two general medicine hospital wards	Environmental cueing/priming	Physical environment	Yes
King et al. 2016 [39]	USA	RCT	Improve hand hygiene compliance	Surgical ICU unit	Environmental cueing/priming	Physical environment	Yes
Orloski et al. 2019 [40]	USA	Prospective controlled pre-post study	Improve patient experience by having providers sit down during examination	Two emergency departments	Environmental cueing/priming	Physical environment	Yes
Meeker et al. 2016 [41]	USA	RCT	Improve guideline concordance for acute respiratory infection treatment	47 primary care practices	Peer comparison; Accountable justification; Suggested alternatives	EHR; Email	Yes
O’Reilly-Shah et al. 2018 [42]	USA	Prospective pre-post study with cross-over	Improve compliance with lung-protective ventilation strategies during general anaesthesia	2 academic hospitals, 2 non-academic hospitals and 2 academic surgery centres	Defaults/pre-orders; Feedback	Medical device/machine; Email	Yes
Kwok et al. 2016 [43]	Australia	Prospective pre-post study	Improve hand hygiene compliance	2 hospital wards (medical and surgical)	Feedback; Goal setting,	Email; Routine activities	No
Patel et al. 2018 [44]	USA	RCT	Increase guideline-concordant statin prescriptions	32 primary care clinics	Alerts/reminders; Active choice; Peer comparison	Email, Electronic dashboard	Yes
Lehmann et al. 2016 [45]	The Netherlands	RCT	Improve influenza vaccination rates of staff	Tertiary care center	Defaults/pre-orders	Email	No
Schmidtke et al. 2019 [46]	England	RCT	Improve influenza vaccination rates of staff	Hospital	Alerts/reminders	Letter	No
Harewood et al. 2011 [47]	Ireland	RCT	Evaluate effect of pre-filling sedation syringes on colonoscopy sedation practices	Endoscopy specialist care	Defaults/pre-orders	Routine activities	Yes
Shakespeare et al. 2018 [48]	Australia	Prospective pre-post study	Improve administration of analgesic medications after Caesarean section	Tertiary teaching hospital	Education; Defaults/pre-orders	Routine activities	Yes
Arora et al. 2019 [49]	USA	Prospective pre-post study	Improve inpatient sleep	2 general medicine hospital wards	Active choice; Environmental cueing/priming; Education	EHR; Physical environment; Routine activities	Yes
Kim et al. 2018 [50]	USA	Retrospective controlled study	Increase influenza vaccination rates and remove any variation due to appointment time	11 primary care clinics	Active choice	EHR	Yes
Patel et al. 2017 [51]	USA	Retrospective controlled pre-post study	Increase influenza vaccination rates	3 primary care clinics	Active choice	EHR	Yes
Malhotra et al. 2016 [52]	USA	Retrospective pre-post study	Increase the prescription of generic medication	Multispeciality practice	Defaults/pre-orders	EHR	Yes
Monsen et al. 2019 [53]	USA	RCT	Reduce prescription of high-cost (low-value) medication	58 primary care and 152 specialty care clinics	Active choice	EHR	Yes
Sedrak et al. 2017 [54]	USA	RCT	Reduce unecessary inpatient tests	3 hospitals	Information transparency	EHR	No
Sharma et al. 2019 [55]	USA	RCT	Reduce imaging tests for patient undergoing palliative radiotherapy	5 radiation oncology practices	Defaults/pre-orders	EHR	Yes
Bourdeaux et al. 2013 [56]	England	Retrospective pre-post study	Increase prescription of chlorhexidine mouthwash and reduce the prescription of hydroxyethylstarch to patients in ICU	Tertiary care ICU unit	Defaults/pre-orders	EHR	Yes
Panattoni et al. 2018 [57]	USA	Retrospective pre-post study	Increase compliance with diabetes preventive care (use of routine glycated hemoglobin)	Ambulatory healthcare system	Alerts/reminders; Defaults/pre-orders	EHR	Yes
Holt et al. 2010 [58]	England	RCT	Improve clinical outcomes and data quality related to cardio-vascular disease	19 primary care clinics	Alerts/reminders	EHR	No

Sixteen of the studies delivered their intervention via Electronic Health Record (EHR) systems, either through the prescription and ordering interface or through patient records. Six studies delivered the intervention mainly through modifications in the physical environment such as posters [36, 37], aromatization [29, 38, 39], or props [40]. Four studies combined other strategies with email communication in order to provide feedback and statistics on the performance of the target behaviour [37, 41–43]. One study utilized email messages to direct participants to an electronic dashboard [44]. One study delivered its intervention solely through an email message [45]. And one additional study delivered its intervention solely through a letter [46].

The two studies targeting modification of ventilation practices included the modification of default setting on the ventilation machines [30, 42] One additional study delivered a cost awareness message printed on test results [31]. Two studies delivered their interventions mainly through the modification of routine activities, namely the modification of syringe sizes for sedation during endoscopy procedures [47], and modification of inpatients’ printed charts with pain management procedures [48].

### Nudging strategies and objectives

**Fig. 3:**
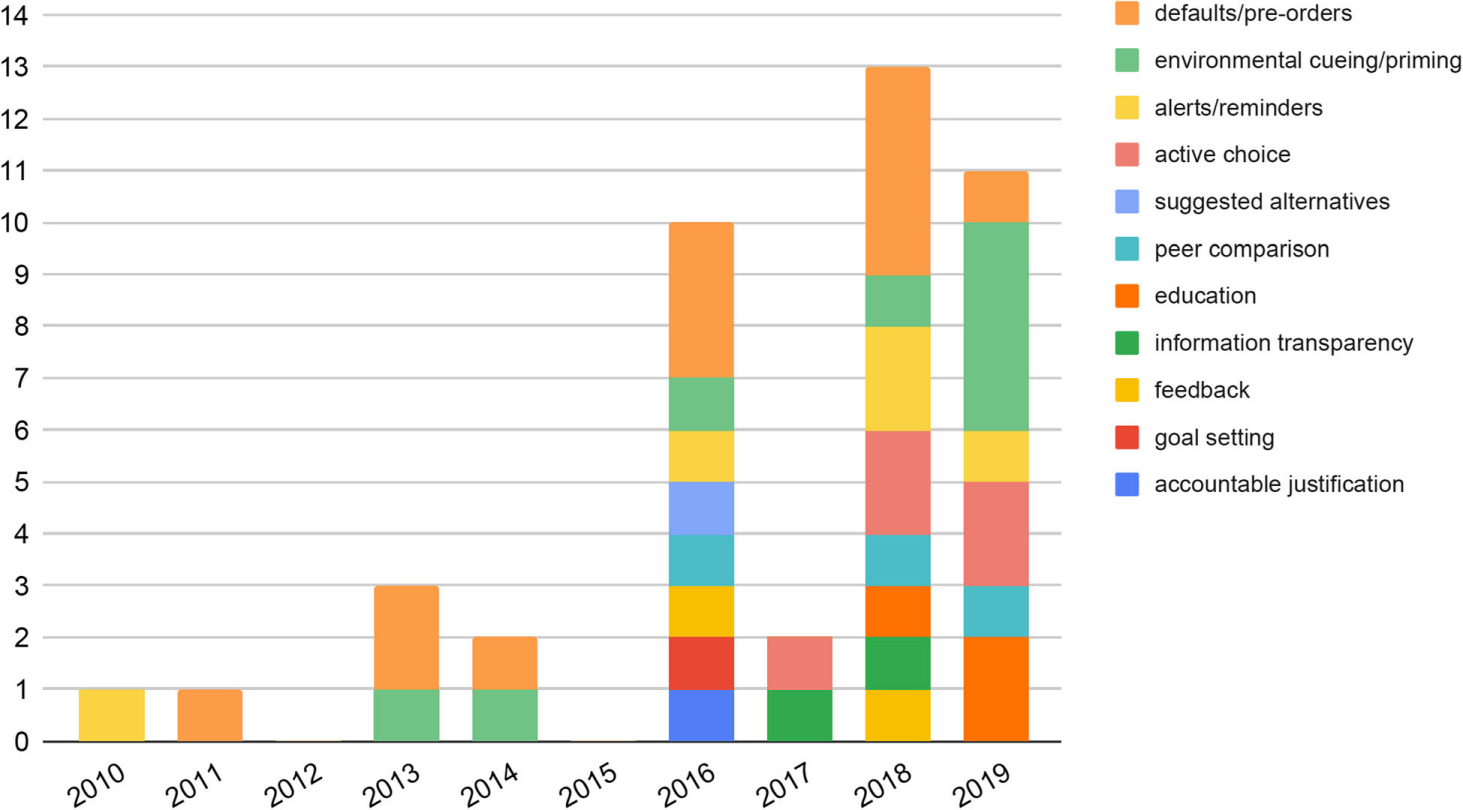
Intervention strategies over time, 2010–2019

In total, 43 nudges were used in the 30 included studies, since 10 studies employed two or three nudging techniques each. From these we identified and coded 11 different nudging strategies, described in Fig. 3 and Table 3. The large majority of studies employed only one nudging strategy (*n* = 20). From 2016 onward, 7 studies used 2 different nudging strategies, and 3 studies included 3 nudging strategies each (See Table 2). All but one of these” multi-nudging” studies had a positive result. Of all nudges, 84% resulted in statistically positive results.

**Table 3:** Description of intervention strategies

Intervention strategy	Description	***N***	Studies
Environmental cueing and priming	Strategies that expose the participant to certain cues (e.g. words, smells, or images) in order to alter behaviour subliminally. These strategies work by activating particular representations or associations in memory just before carrying out the target behaviour	8	[30, 32, 37–41, 50]
Defaults and pre-orders	Strategies by which the default option is chosen so as to minimize or facilitate the path to the desired behaviour, for example, making all prescriptions based on generic medication and requiring additional actions to prescribe a branded medication, or having participants opt-out instead of opt-in to a desired behaviour. We have also included in this category all user-interface design strategies for electronic systems that aim to make a given behaviour more likely such as showing recommended actions more prominently, or purposefully grouping certain sets together	12	[31, 34–36, 43, 46, 48, 49, 53, 56–58]
Suggested alternatives	Strategies that automatically detect a certain behaviour and immediately suggest an alternate course of action, typically within EHR systems. We have differentiated these from Default strategies because they intrinsically include a dialog with the user, where the user is prompted to critically analyse options and make an informed decision.	1	[42]
Active choice	A strategy that prompts the user to make an immediate decision, for example, a dialog box opens when a patient record is being accessed and asks the provider to accept or reject a vaccination order. Active choice is different from suggested alternatives in that the trigger for the choice often comes from the system and not the user.	5	[45, 50–52, 54]
Alerts and reminders	Prompts from the system that warn the user of an event of interest, for example, an expensive medication order or a ventilation setting outside recommended limits. Unlike active choice, alerts do not necessarily present an immediate choice to be taken in the system.	5	[31, 45, 47, 58, 59]
Accountable justification	A strategy where any choice other than the recommended choice must be justified, often with a text entry. This requires a critical analysis of why that choice was made.	1	[42]
Information transparency	Strategy by which relevant information is somehow presented to the user, for example, adding the cost of a test next to the name of the test in the ordering system. Information transparency does not require any specific action from the user and the user may or may not be aware of the information shown.	2	[33, 55]
Feedback	The communication of the frequency of the target behaviour back to the user. It may be an aggregated statistic such as average compliance in the department, or it may be personalized to the participant such as the number of antibiotics prescribed in the period of interest.	2	[43, 44]
Peer comparison	A specific form of feedback where the participant is compared with other colleagues, either anonymously or transparently.	3	[38, 42, 45]
Goal setting	Strategy often combined with feedback where the participants are prompted to set a target behaviour and they follow-up on that goal.	1	[44]
Education	Not traditionally seen as a nudging strategy, but we included it as a strategy since some of the articles complemented their intervention with an educational information session.	3	[41, 49, 50]

Since outcomes were very heterogeneous, we clustered articles into four different objectives to facilitate comparison. The majority of studies had as their objective to change prescription and ordering behaviour, namely, encouraging judicious antibiotic prescription [34, 36, 37, 41], increasing vaccinations orders [49, 50], increasing prescription of generic medication [51], reducing prescription of high-cost, low-value medication [32, 52], reducing unnecessary laboratory tests [33, 53], reducing imaging procedures [31, 54], increasing prescription of mouthwash to intubated patients in the intensive care unit (ICU) [55], increasing guideline-concordant prescription of statins [44], increasing prescription of blood glucose (A1C) tests for diabetes prevention [56], and increasing high-value treatment for lower-back pain [35].

The second largest target was the modification of behaviours with respect to certain care procedures (*n* = 7) such as ventilation settings for intubated patients [30, 42], improving inpatient sleep [49], sedation during endoscopy [47], screening for risk of cardio-vascular disease in primary care [58], sitting down during examinations [40], and pain management after Caesarean Section surgery [48]. Four studies targeted hand hygiene [29, 38, 39, 43], and two studies focused on vaccination of healthcare providers [45, 46].

Of the 17 studies with the objective of changing prescription and ordering behaviour all but two had a successful outcome (statistically significant positive results). Of the 7 studies targeting the modification of behaviours with respect to certain care procedures, only one had a non-significant result. Of the 4 studies targeting hand hygiene, three were successful. Of the 2 studies targeting vaccination of healthcare providers none had a positive result.

### Quadrants

During the analysis, we found that some strategies appealing to the analytical System 2 could only cause impact if the participant chose to pay attention. For example, only showing the cost of a particular medication on the ordering screen (information transparency) can go completely unnoticed by a participant thereby not engaging the participant’s analytical mind. We also found that some strategies that automatically substituted brand medications for generics or automatically populated ordering forms (defaults/pre-orders) could also take place without the participant’s knowledge or awareness. Thus, these interventions could change the outcome of a certain behaviour without having any effect on the behaviour itself.

Another challenge with considering System 1 and System 2 categories is that they do not capture whether the intervention is presented at the time of the decision or not. For example, some interventions sent feedback emails that could be read anytime, not at the time of prescription. So, the emails are meant to change a belief that will eventually impact a behaviour, but that is very different from immediately changing a prescription to generic at the time of ordering. To capture these shortcomings, we instead developed two independent dimensions, creating four quadrants:

- Synchronous vs. asynchronous - An intervention strategy was coded as *synchronous* if its delivery coincided with the decision or behaviour it intended to affect.
- Active vs. passive - An *active* strategy cannot be completed without an action on the part of the participant.

**Fig. 4:**
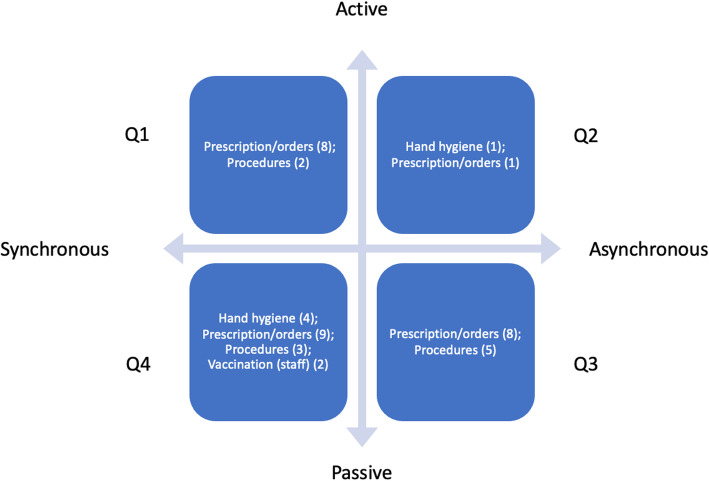
Intervention objectives across quadrants

Figure 4 shows the distribution of nudging objectives across the quadrants. With the exception of staff vaccination, the other nudging objectives were addressed by strategies in more than one quadrant.

**Fig. 5:**
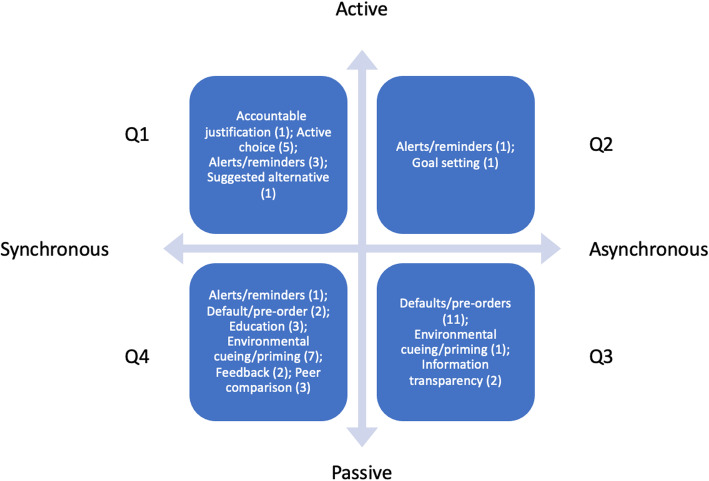
Nudging strategies across quadrants

Figure 5 shows the distribution of intervention strategies across the quadrants. Three intervention strategies were assigned to more than one quadrant: alerts/reminders, defaults/pre-orders, environmental cueing/priming. Alerts and reminders often take place when the target behaviour must be performed (synchronous) and require an action from the clinician (active). Two exceptions were observed. One study was coded as active and asynchronous (Q2) [44] as it sent an email reminder prompting clinicians to access a dashboard in order to be exposed to the other nudges. The other study [46] was coded as passive and asynchronous (Q3), where letters were sent to remind front line staffers to get vaccinated, the letters could be read at any time and no action was strictly required.

Most of the environmental cueing/priming strategies are passive and asynchronous (Q3), for example, placing posters with crafted messages and pictures. However, we coded one study [40] as synchronous because it placed chairs in examination rooms to encourage participants to sit during examination (Q4). In this case, the priming object (chair in examination room) coincides in time with the target behaviour (sitting during examination). The majority of default/pre-order interventions were synchronous and passive (Q4), for example, automatically changing a brand name medication for its generic equivalent. The” nudge” (substituting for generic) coincided in time with the target behaviour (prescribing a medication), and happened automatically even if there was no action from the clinician. There were two exceptions in this category, which implemented asynchronous strategies. One study [56] periodically populated an electronic folder with pre-approved orders for A1C laboratory tests, the other study [45] sent letters to participants with either pre-booked appointments (opt-out) for vaccination or without pre-booked appointments (opt-in).

## Discussion

Since the term” nudge” was introduced already in 2008, and gained much attention as a viable intervention strategy for behavioural change, it was surprising that only 30 studies could be included in the review. However, the majority of included records were published in the last 4 y of our inclusion period. This could possibly indicate that nudging is becoming more popular in the context of behaviour interventions for HCP. Our result is in line with a new literature review addressing studies that are explicit about their use of nudge theory in influencing clinician behaviour, but with a slightly different inclusion criteria making our study complementary [59]. The authors of this review identified only 22 studies of relevance over a 10-year period (2008–2018) conducted in multiple settings and contexts.

Very few articles actually use the term “nudge” in their title or abstract, which was a necessary requirement for inclusion. Interestingly, the term “nudge” is often used in commentaries and editorials, but not in scientific articles. Rather than describing an actual nudging intervention, several articles instead discuss the potential of nudging for public policy or its ethical implications, something that has also been observed by others [59, 60].

In total, 43 nudges were used in the 30 included studies, since 10 studies employed two or three nudging techniques each. All but one of these” multi-nudging” studies had a positive result. Of all nudges, 84% resulted in statistically positive results. We included education – not traditionally seen as a nudging strategy – as a strategy since three of the” multi-nudging” articles complemented their intervention with an educational information session [40, 48, 57]. The combination of several intervention strategies, on the one hand, seems to increase the possibility that an intervention is effective, but on the other hand, makes it hard to determine exactly which elements make an intervention successful. In total, all but six studies had a positive result, which was very encouraging.

We would here like to discuss two of the target behaviours considered in our review: judicious antibiotic prescribing, and hand hygiene. All four studies targeting judicious antibiotic prescription [34, 36, 37, 41] had positive outcomes. This is an important finding since inappropriate antibiotic prescription increases cost of care, causes adverse drug reactions, and affects the growth of antibiotic-resistant bacteria [61–63]. Of these four interventions, two posted commitment letters in examination rooms (priming) [36, 37], two studies employed peer comparison over email messages [37, 41], and one study changed the default presentation of treatment options in an EHR [34]. One study [41] combined accountable justification with suggested alternatives via EHR by warning clinicians that “antibiotics are not recommended for this diagnosis. See alternatives below” and requiring that clinicians give a written explanation for prescribing antibiotics.

Hand hygiene is considered one of the most important measures to prevent healthcare-associated infections [64–66]. Three out of four of the interventions targeting hand hygiene were successful [29, 38, 39], employing priming and environmental cueing strategies. These interventions dispersed scents permeating the environment [29], displayed posters in the vicinity of gel dispensers with carefully crafted messages [39], or showed a pair of male eyes [39] in order to successfully increase hand hygiene compliance. Both the visual and olfactory cues described above were passive and asynchronous. The non-successful intervention [44] used goal setting and peer pressure instead, being active and asynchronous. But despite not achieving a significant positive result, the study showed that social cohesion – colleagues reminding each other to wash their hands – could improve hand hygiene.

Given the low cost of these priming interventions, they should be considered as viable strategies, especially in view of the recent covid-19 pandemic, when hand hygiene is a public health concern.

As noted by Sunstein [3] default rules may well be the most effective nudge, and this seems also to be the case when it comes to nudging clinicians [59]. This can be explained by status quo bias [67] and decision inertia [68], meaning that we rather keep things as they are.

One could argue, however, that creating default processes which do not require any action from the clinician may pass unnoticed. For example, an electronic prescribing system may automatically change a brand medication for its generic equivalent in a subtle way. This raises concern over infringing ethical principles to nudge someone being unaware of the nudge [2, 69–73]. Sunstein argues that unless active choosing is involved, some kind of default rule is essentially inevitable, regardless of whether it is intentional or not [3]. Hence, it is crucial that this type of nudge is clearly observed and that there is an opt out so that professionals can reverse the nudge to their original preference. The default studies in our review all included the possibility for the HCP to override the default, for instance prescribing another drug or changing ventilator settings. This highlights the importance of transparency, but also as suggested by Hofmann & Stanak [73] that the means of nudging also have to be in proportion to the benefit-harm ratio.

We found that, after default nudges (*N* = 7), active choice was the second most common strategy (*N* = 5) to affect prescribing behaviour. In contrast to an unnoticed default nudge, the active choice brings the choice to the forefront and requires a thoughtful action from the clinician.

### Limitations

Our study has several limitations. First, we did not search other databases outside medicine and healthcare. Our rationale for this was to focus on the use of nudging strategies to affect healthcare professionals with potential effects for clinical practice. The downside of this decision is that we risk missing potentially important studies not included in PubMed and PsycINFO.

Second, we chose the terms” nudge” or” nudging” or” nudges” as our point of departure. Our intention was to find research that specifically referred to the nudging theory proposed by Thaler and Sunstein in 2008 and to better understand how it was being interpreted and employed to affect HCP in clinical settings. We chose our search terms to reflect what we believed were the most commonly known and broadly inclusive terms for describing their theory. In doing so however, we might have missed many relevant articles using related terms like “choice design” and “choice architecture”. We will include more related terms in future research.

Third, this focus on “nudging” leaves out many other interventions for quality improvement in clinical settings. We recognize that the space of possible behavioural change interventions for quality improvement in clinical settings includes many different theories other than nudging. In this article, however, we wanted to understand how nudging, as described by Thaler and Sunstein, is affecting the design of interventions for HCP.

Fourth, the heterogeneity of the articles included and the lack of consensus regarding a theoretical framework around nudging made it quite challenging for us to identify and code the relevant dimensions of the interventions. In our attempt to better categorize the interventions we introduced even newer terms that will have to be scrutinized in future work. That said, we find that the synchronous/asynchronous and active/passive dimensions provide a practical way for non-experts to reason about nudging interventions and how they may be implemented in a clinical setting.

## Conclusion

The purpose of this scoping review was to identify how nudging, as described by Thaler and Sunstein, is influencing the design of interventions to affect HCPs in clinical settings. This is one of the first reviews to consider nudging as a general strategy to affect the behaviour of healthcare professionals, not limited to particular application areas nor a specific type of intervention. Despite the popularity of the term” nudging” in editorials and commentary, interventions do not often allude to nudging as defined by Thaler and Sunstein in 2008 [1]. We identified only 30 articles which mentioned terms related to” nudging” in the title or abstract and described an intervention targeting the behaviour of HCPs in a clinical setting.

When trying to assess and compare interventions with very different objectives and intervention strategies, we found that Dual Process Theory [11] or System 1 and System 2 thinking [10] did not provide a sound basis for the characterization of the interventions. It was very difficult to determine whether the nudging strategy employed was targeting System 1 or System 2, or even if the rationale for the strategy was truly having the intended effect on the clinician. For example, showing the cost of a medication can only prompt the clinician to reason about cost if he or she: (i) sees this information and (ii) decides to consider it. We therefore created two more practical dimensions to characterize nudging strategies: (a) passive/active, and (b) synchronous/asynchronous. In particular, we found that the active/passive dimension had important implications for whether the intervention was changing a behaviour long term or simply biasing a specific outcome in a given moment, and whether it infringes on any ethical principles to influence someone’s behaviour when they are not aware of the” nudge”. It is imperative that nudging is addressed in an explicit and transparent manner [73] and we therefore argue that the active/passive dimension can be used in practice to evaluate and design ethically sound nudging interventions for HCPs.

### Implications for research

More research is needed on the impact of nudging healthcare providers’ attitudes and behaviours. Our study shows a large set of different nudging strategies and provides a basic yet practical categorization to explain and compare the mechanisms of different strategies. Careful ethical consideration should be given to passive strategies of which the user might not be aware. Alternatively, active strategies present fewer ethical dilemmas and are better suited for explaining the impact of nudging interventions in the behaviour of healthcare providers in the short and long term.

## Supplementary Information

**Additional file 1.**

**Additional file 2.**

## Data Availability

The datasets supporting the conclusions of this article are included within the article (and its additional files).

## Abbreviations

A1C: Glycated haemoglobin, or glycohemoglobin test
DPT: Dual Process Theory
EHR: Electronic Health Record
HCP: Healthcare professional
ICU: Intensive Care Unit
Q1: Active and synchronous
Q2: Active and asynchronous
Q3: Passive and asynchronous
Q4: Passive and synchronous
RCT: Randomized Controlled Trial
