# Supplementary material for: Nudging healthcare professionals in clinical settings: a scoping review of the literature
Source: BMC Health Serv Res. 2021 Jun 2;21:543. doi: 10.1186/s12913-021-06496-z (PMC8170624; doi:10.1186/s12913-021-06496-z)
Supplement: Supplementary file 2 — Additional file 2. [file 12913_2021_6496_MOESM2_ESM.pdf]

Literature search

Datum: 2019-09-23

Search question: How is nudging being used to affect the behavior of healthcare professionals in a clinical setting?
Language: Engelska
Period: 2010 och framåt

Results: 1024 referenser
Databases used: PubMed, PsycINFO
Search strategy: see page 2

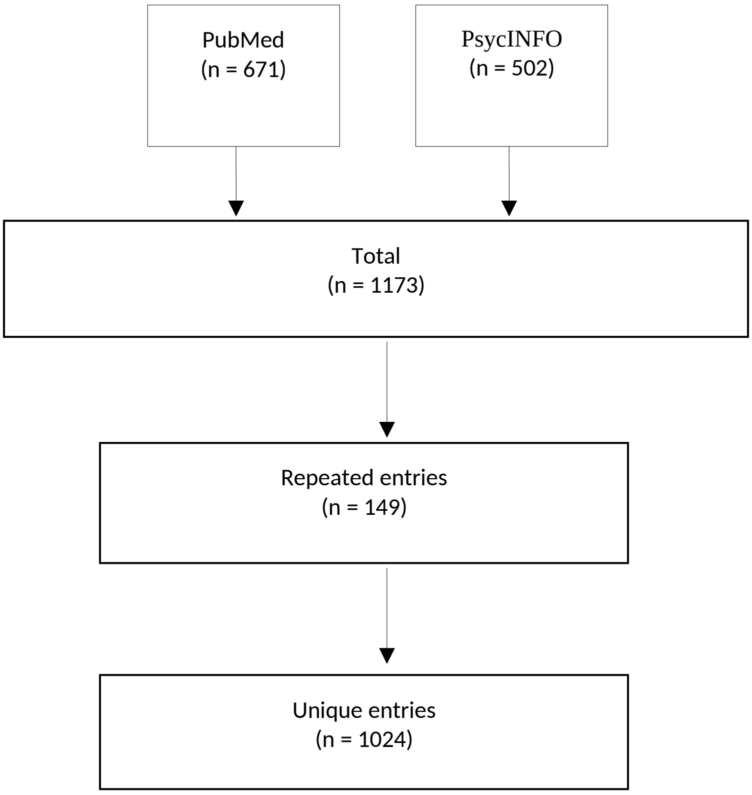

Database: PubMed
Date: 2019-09-23
No of results: 671 ref

Searc h	Add to builder	Query	Items found
#3	Add	Search **nudges OR nudging OR nudge** Filters: **Publication date from 2010/01/01; English**	**671**
#2	Add	Search nudges OR nudging OR nudge Filters: Publication date from 2010/01/01	680
#1	Add	Search nudges OR nudging OR nudge	759

Database: PsycINFO
Date: 2019-09-23
No of results: 502 ref

0	

#	Undran	Avgränsare/Utökning	Senast körd via	Result at
**S3**	**TI (Nudges OR Nudging OR Nudge) OR AB (Nudges OR Nudging OR Nudge) OR KW (Nudges OR Nudging OR Nudge)**	**Avgränsare - Publikationsdatum: 20100101-20191231; Engelska**	**Gränssnitt - EBSCOhost Research Databases**<br><br>**Databas - PsycINFO**	**502**
S2	TI (Nudges OR Nudging OR Nudge) OR AB (Nudges OR Nudging OR Nudge) OR KW (Nudges OR Nudging OR Nudge)	Avgränsare - Publikationsdatum: 20100101-20191231	Gränssnitt - EBSCOhost Research Databases<br><br>Databas - PsycINFO	511
S1	TI (Nudges OR Nudging OR Nudge) OR AB (Nudges OR Nudging OR Nudge) OR KW (Nudges OR Nudging OR Nudge)		Gränssnitt - EBSCOhost Research Databases<br><br>Databas - PsycINFO	605

Medicinska biblioteken
Sahlgrenska Universitetssjukhuset
www.sahlgrenska.se/bibliotek
